# Lipid Metabolism in Breast Cancer: From Basic Research to Clinical Application

**DOI:** 10.3390/cancers17040650

**Published:** 2025-02-14

**Authors:** Xiangyu Huang, Bowen Liu, Songjie Shen

**Affiliations:** 1 Department of Breast Surgery, Peking Union Medical College Hospital, Chinese Academy of Medical Sciences and Peking Union Medical College, Beijing 100032, China; huangxia@student.pumc.edu.cn (X.H.); liubowen@pumch.cn (B.L.); 2 School of Life Sciences, Tsinghua University, Beijing 100084, China; 3 Ambulatory Medical Center, Peking Union Medical College Hospital, Chinese Academy of Medical Sciences and Peking Union Medical College, Beijing 100032, China

**Keywords:** breast cancer, lipid metabolism, reprogramming, targeting therapy

## Abstract

Breast cancer remains the most prevalent cancer among women globally, with significant links to obesity and lipid metabolism abnormalities. This review examines the role of lipid metabolism in breast cancer progression, highlighting its multifaceted contributions to tumor biology. We discuss key metabolic processes, including fatty acid metabolism, triglyceride metabolism, phospholipid metabolism, and cholesterol metabolism, detailing the reprogramming that occurs in these pathways within breast cancer cells. Alterations in lipid metabolism are emphasized for their roles in supporting energy production, membrane biogenesis, and tumor aggressiveness. Furthermore, we examine how lipid metabolism influences immune responses in the tumor microenvironment, affecting immune cell function and therapeutic efficacy. The potential of lipid metabolism as a target for novel therapeutic strategies is also addressed, with a focus on inhibitors of key metabolic enzymes. By integrating lipid metabolism with breast cancer research, this review underscores the importance of lipid metabolism in understanding breast cancer biology and developing treatment approaches aimed at improving patient outcomes.

## Simple Summary

Lipid metabolism exerts a pivotal function in various types of cancers, including breast cancer. Currently, the significance of lipid metabolism in supporting energy production, membrane biogenesis, and tumor aggressiveness has been acknowledged by the scientific community. However, further investigations are required to explore the reprogramming and regulatory pathways associated with this process. This review summarizes current research progress in lipid metabolism in breast cancer and addresses the potential of lipid metabolism as biomarkers and therapeutic targets, which helps to elucidate breast cancer biology and develop treatment approaches aimed at improving patient outcomes.

## 1. Introduction

According to data from the International Agency for Research on Cancer, breast cancer constituted 11.7% of all new tumor cases and 6.9% of cancer-related deaths among women in 2020, making it the most prevalent cancer globally [1]. In recent years, obesity has emerged as a significant risk factor for breast cancer, particularly due to its association with enhanced adiposity [2]. This correlation highlights a crucial role for lipid metabolism in the development and progression of breast cancer, a notion supported by a growing body of clinical evidence. Notably, research has indicated that patients with metabolic syndrome, which is characterized by obesity and elevated blood lipid levels, are more prone to developing triple-negative breast cancer (TNBC) [3]. Moreover, studies have also indicated that elevated plasma levels of both high-density lipoprotein (HDL) and low-density lipoprotein (LDL) are associated with a higher risk of breast cancer [4]. These findings highlight the crucial role of lipid metabolism disruptions in breast cancer research and suggest that further assessment of lipid-related pathways could lead to innovative prevention and treatment strategies. As the understanding of the role of lipid metabolism in breast cancer deepens, new avenues for targeted therapies are opened that could improve patient outcomes.

Lipids are hydrophobic or amphiphilic molecules derived from carbanion-based condensation of thioesters and/or carbocation-based condensation of isoprene units [5]. They play diverse roles in biological systems, including the structural organization of cellular membranes, energy storage, and signal transduction [6]. The dynamic nature of lipid metabolism allows for the reprogramming of lipid profiles, which is critically significant in breast cancer progression. Specifically, alterations in lipid metabolism can influence tumor growth, metastasis, and cancer microenvironment in breast cancer [7]. To further unveil the complex lipid-related processes of cancer development, a plethora of tools for studying lipids has emerged in recent years, with lipidomic tools being of significant importance. Lipidomics comprises the comprehensive analysis and identification of lipids, aiming to elucidate the intricate mechanisms of lipid metabolism. By integrating lipidomics with breast cancer research, scientists are better positioned to uncover the multifaceted roles of lipids in tumor biology, ultimately paving the way for novel therapeutic strategies.

## 2. The Process of Lipid Metabolism and Relevant Research Methods

### 2.1. Brief Introduction of Lipid Metabolism

**Figure 1:**
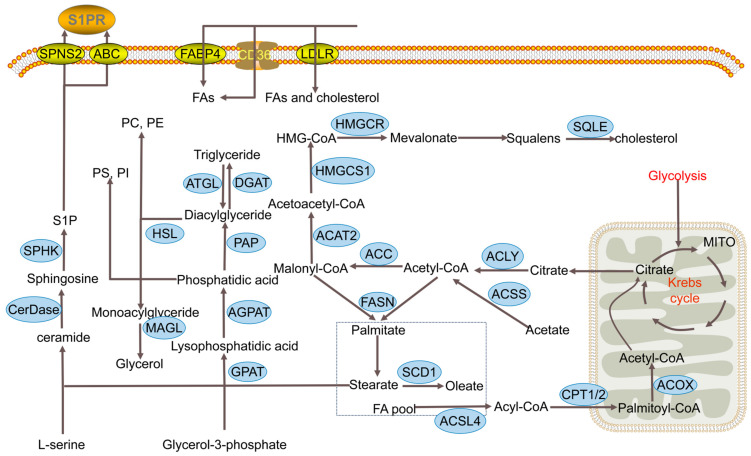
Overall view of lipid metabolism in breast cancer cells. This figure shows the key intermediates and enzymes involved in lipid metabolism pathways. The abbreviations in this figure are shown in the ‘Abbreviations’ section.

Lipid metabolism refers to the series of chemical processes whereby lipids are synthesized, broken down, absorbed, and modified to facilitate the processing of substances essential for maintaining normal physiological functioning. The lipid metabolism process can be divided into four parts: fatty acid metabolism, triglyceride metabolism, phospholipid metabolism, and cholesterol metabolism (Figure 1). In the following sections, each part of the lipid metabolism process will be discussed separately.

#### 2.1.1. Fatty Acid Metabolism Process

Fatty acid metabolism can be further subdivided into three distinct processes: de novo synthesis of fatty acids, fatty acid uptake, and fatty acid oxidation (FAO). De novo synthesis of fatty acids begins with acetyl-CoA, which is first converted into citrate in the mitochondria through the tricarboxylic acid cycle (TCA). Citrate is then transported out of the mitochondria and reconverted into acetyl-CoA in the cytoplasm by ATP citrate lyase (ACLY) [8]. In addition to ACLY, acetyl-CoA synthetase (ACSS) also catalyzes acetyl-CoA synthesis within the cytoplasm [9]. Acetyl-CoA in the cytoplasm is then converted to malonyl-CoA by acetyl-CoA carboxylase (ACC), which serves as the rate-limiting step in de novo fatty acid biosynthesis. One acetyl-CoA molecule and seven malonyl-CoA molecules can then interact to form palmitic acid under the catalysis of fatty acid synthase (FASN). Palmitic acid can be further elongated by fatty acid elongases or desaturated by fatty acid desaturases, including stearoyl-coenzyme A desaturase 1 (SCD1), thereby enhancing the variety of fatty acids [10].

Exogenous fatty acids, mainly derived from dietary lipids, are also important sources of fatty acid acquisition. Free fatty acids can be transported into cells via cluster differentiation 36 (CD36) or other fatty acid transport proteins (FATPs) [11]. This process is aided by fatty-acid-binding proteins (FABPs) [12]. LDL also contains polyunsaturated fatty acids (PUFAs), whose uptake can be achieved via LDL receptor (LDLR)-mediated endocytosis [13].

FAO is a critical source of energy for cells. Fatty acids are first converted into fatty acyl-CoAs by fatty acyl-CoA synthetase (ACSL), which activates the FAO pathway. To cross the mitochondrial membrane, fatty acyl-CoAs must be modified by carnitine palmitoyl transferase 1 (CPT1) and are then regenerated as fatty acyl-CoA in the mitochondrial matrix under the catalysis of carnitine palmitoyl transferase 2 (CPT2) [14]. Once inside the mitochondrial matrix, fatty acyl-CoAs undergo a repeated β-oxidation process, which consists of four steps: dehydrogenation, hydration, dehydrogenation again, and thiolysis. Acyl-CoA oxidase (ACOX) is a rate-limiting enzyme in β-oxidation of branched and long-chain fatty acids. Each cycle of β-oxidation releases one molecule of acetyl-CoA, which then enters the TCA cycle in the mitochondria to supply energy for cells [15].

#### 2.1.2. Triglyceride Metabolism Process

The triglyceride metabolism process involves two parts: triglyceride synthesis and triglyceride breakdown. Triglyceride synthesis involves two distinct pathways: the phosphoglyceride and the monoacylglyceride (MAG) routes, with the phosphoglyceride route being the most prominent. In this route, glycerol phosphate undergoes a series of reactions with fatty acyl-CoA molecules, catalyzed by specific enzymes. Initially, it reacts with a molecule of fatty acyl-CoA to produce lysophosphatidic acid (LPA) under the catalysis of glycerol-3-phosphate O-acyltransferase (GPAT), which is then catalyzed by acylglycerol-3-phosphate O-acyltransferase (AGPAT) to form phosphatidic acid (PA). Phosphatidic acid is converted into diacylglyceride (DAG) via catalysis by phosphatidyl phosphatases, e.g., LPIN1. Subsequently, DAG reacts with a molecule of fatty acyl-CoA to produce triglycerides, a process that is catalyzed by diacylglycerol o-acyltransferase (DGAT) [16,17].

Triglyceride breakdown is an almost reverse process of triglyceride synthesis. Under continuous catalysis of adipose triglyceride lipase (ATGL), hormone-sensitive lipase (HSL), and monoacylglyceride lipase (MAGL), triglycerides release one molecule of fatty acid successively [16]. One molecule of triglyceride eventually breaks down to produce one molecule of glycerol and three fatty acid molecules, which serve as material and energy sources for breast cancer cells.

#### 2.1.3. Phospholipid Metabolism Process

There is significant overlap between phospholipid and triglyceride synthesis pathways. The core reaction of both pathways is the condensation of glycerol (or sphingosine) with fatty acids. Many enzymes, including GPAT, LPIN, MAGL, and so on, are involved in both pathways, whose metabolism has been described above. However, the synthesis of phospholipids additionally needs the incorporation of supplementary moieties, according to which phospholipids can be divided into glycerophospholipids (GPLs) and sphingolipids (SPLs).

The synthesis of the GPL family mainly involves two pathways. Phosphatidylserine (PS) and phosphatidylinositol (PI) can be derived from PAs, while phosphatidylcholine (PC) and phosphatidylethanolamine (PE) can be derived from DAGs. These GPLs are constituent elements of compositional diversity of biological membranes. GPLs can be degraded by a series of phospholipases which act on different ester bonds, generating a variety of products involved in other lipid metabolism processes [18].

SPLs contain the long-chain amino alcohol sphingosine, esterified to a fatty acid and a phosphate group [19]. Ceramide (Cer) is considered the central hub of SPL metabolism, which can be synthesized through several pathways, including the de novo pathway, the sphingomyelin pathway, and the salvage pathway [20]. Many enzymes, including L-serine:palmitoyl-CoA transferase (SPT), ceramide synthase (CerS), and dihydroceramide desaturase (DES), are involved in ceramide synthesis pathways. Cers can be converted into various types of SPLs, especially being degraded by ceramidases (CDases) to produce sphingosines, which are then phosphorylated by sphingosine kinase 1/2 (SPHK1/2) to generate sphingosine-1-phosphate (S1P), the central bioactive molecule [21]. S1P can be secreted by the S1P transporter spinster homolog 2 (SPNS2) or ATP-binding cassette transporters (ABC), then interacts with S1P receptors 1–5 (S1PR1–5) and activates them in an autocrine or paracrine manner [22,23,24,25].

#### 2.1.4. Cholesterol Metabolism Process

The metabolism of cholesterol comprises two principal processes: the synthesis of cholesterol and its uptake and secretion. The mevalonate pathway is the process of cholesterol synthesis. In total, three molecules of acetyl-CoA are thiolized, then combine to form hydroxymethylglutaryl-CoA (HMG-CoA) under the catalysis of HMG-CoA synthase (HMGCS). Next, HMG-CoA is reduced to form mevalonate by HMG-CoA reductase (HMGCR) [26]. Mevalonate is activated by ATP and then condensed to form squalene. Lanosterol is produced by epoxidation of squalene, which is catalyzed by squalene cyclooxygenase (SQLE). Lanosterol is finally converted to cholesterol in the presence of oxygen and NADPH [10]. As with the uptake of fatty acids, cholesterol uptake can be accomplished through the function of LDLR. Cholesterol levels are also influenced by cholesterol secretion through the export of lipoproteins.

### 2.2. Advances in Lipid Metabolism Research Techniques

The pivotal roles of lipids in numerous biological functions make investigation of lipid metabolism reprogramming an invaluable approach to elucidate the complex mechanisms underlying diverse pathologies, including cardiovascular diseases, diabetes, and cancers. Therefore, methods for identifying and quantifying lipids warrant significant attention in this field. The latest advances in science and technology have led to continual refinement and enhancement of research techniques pertaining to lipid metabolism. These techniques can be divided into three parts: sample separation and purification, sample screening, and quantitative analysis.

Sample separation and purification are crucial steps in the preparation of lipid metabolism analysis. Early studies mainly employed solvent extraction for lipid purification, while liquid chromatography (LC) has become the predominant means with the advent of chromatographic technology. Recently, many cutting-edge techniques, e.g., ultraperformance liquid chromatography, high-resolution liquid chromatography, and solid phase extraction, have been developed to produce samples with high enrichment and low contamination [27,28]. Technological advances in sample screening have seen a shift from cell-culture-based to biochip-based approaches. The advent of biochip technology has enabled the development of high-throughput screening in vitro, greatly improving the efficiency of lipid metabolism analysis [29]. Quantitative analysis of lipid metabolism is the most fundamental aspect of lipid metabolism research. It is undergoing a significant transition from traditional chemical methods to modern, mass spectrometry (MS)-based techniques. Compared with traditional analytical methods, MS has superior sensitivity, selectivity, and specificity. Its analytical capabilities have increased with advances in MS precision, and more importantly, the evolution of pre-separation methodologies. In recent years, supercritical fluid chromatography and ion mobility spectrometry, as two novel separation techniques, have shown advantages in elution speed and resolution [30,31]. The combination of these techniques has the potential to integrate the advantages of each approach, leading to more robust and insightful results. This is particularly evident in the case of LC-MS, which has become a widely applied tool in modern lipid metabolism research.

Recent advances in innovative technologies, especially single-cell lipidomics, have greatly facilitated the exploration of lipid metabolism. These cutting-edge techniques have enabled researchers to obtain high-resolution lipid profiles, providing new directions and substantial support for the investigation of lipid metabolism in breast cancer. By integrating lipidomics with breast cancer research, scientists are better positioned to uncover the multifaceted roles of lipids in tumor biology, ultimately paving the way for novel therapeutic strategies.

## 3. Lipid Metabolism Reprogramming in Breast Cancer

**Figure 2:**
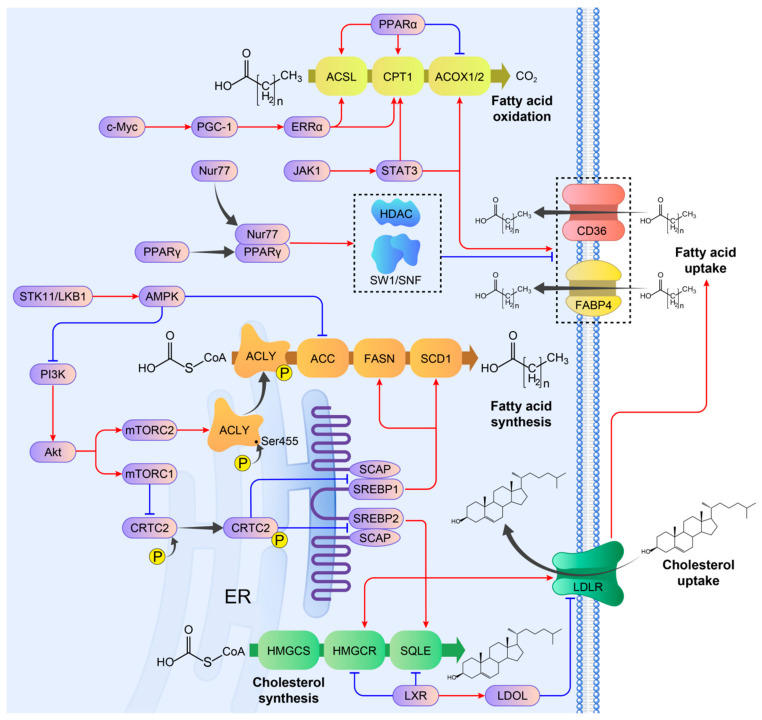
Main regulatory networks of lipid metabolism in breast cancer. The abbreviations in this figure are shown in the ‘Abbreviations’ section.

The growing understanding of breast cancer and the emerging field of lipid metabolic research have led to an indisputable clarification of the interrelationship between abnormal lipid metabolism and breast cancer progression. However, given the intricate nature of lipid metabolism, the involvement of lipid metabolic pathways in breast cancer and the underlying driving mechanisms warrant additional research. In recent studies, lipid metabolism remodeling and the associated regulatory pathways in breast cancer have gradually been revealed, as partially demonstrated in Figure 2. These findings provide further insights into the mechanisms underlying breast cancer development.

### 3.1. Fatty Acid Metabolism Reprogramming in Breast Cancer

#### 3.1.1. De Novo Fatty Acid Biosynthesis Reprogramming

Upregulation of de novo fatty acid synthesis is a hallmark of many malignancies, including breast cancer [32]. In healthy human tissues, fatty acids are mainly derived from dietary lipids rather than de novo synthesis [33]. However, in breast cancer cells, the proportion of de novo synthesized fatty acids is significantly elevated, predominantly consisting of saturated and monounsaturated fatty acids, while PUFAs are noticeably lacking [34]. PUFAs can be easily oxidized by free radicals generated by the labile iron pool via the Fenton reaction, which eventually leads to cell ferroptosis. In turn, upregulation of de novo fatty acid synthesis can enhance the metabolic activity of breast cancer cells and diminish the impact of free radical damage on these cancer cells [35].

Several studies have highlighted enzymes involved in de novo fatty acid biosynthesis, including ACLY, ACC, FASN, and SCD1, which are upregulated in breast cancer (Figure 1). For instance, Chen et al. reported a marked elevation in ACLY expression at both mRNA and protein levels in breast cancer tissues, with high ACLY levels correlating with worse clinical outcomes and resistance to multiple antitumor drugs [36]. In another study, overexpression of ACLY in breast cancer cells increased the expression of the snail protein, which ultimately induced tumorigenesis and cancer stemness [37]. Numerous studies have also indicated that ACC is highly expressed in breast cancer tissues, with its maintenance being crucial for the survival of breast cancer cells [38,39]. Overexpression of FASN was observed in 70% of patients with operable triple-negative breast cancer, which was linked to poor prognosis [40]. Ferraro et al. revealed a strong correlation between FASN expression and brain metastasis in breast cancer [41]. Additionally, SCD is also an upregulated gene in breast cancer tissue, as revealed by quantitative real-time PCR [42]. Moreover, SCD1 overexpression was shown to correlate with poor prognosis in breast cancer patients [43]. Overall, there is a clear upregulation of de novo fatty acid synthesis in breast cancer, meeting the increased demands for membrane biogenesis, energy supply, and signaling pathways in cancer cells.

Furthermore, aberrant regulation of specific signaling pathways in breast cancer can result in abnormal de novo fatty acid synthesis, on which previous scholars have focused and made significant efforts (Figure 2). PI3K/Akt/mTOR signaling is considered a prominent regulatory pathway of de novo synthesis of fatty acids with two regulatory elements, including mTOR complex 1 (mTORC1) and mTORC2. mTORC1 phosphorylates and inactivates cAMP response-element-binding protein (CREB)-regulated transcriptional coactivator 2 (CRTC2), thereby relieving its inhibition on sterol regulatory-element-binding transcription protein 1 (SREBP1) and enhancing ER–Golgi trafficking of the SREBP1/SCAP complex [44,45]. SREBP1, a key transcription factor that regulates de novo synthesis of fatty acids, is overexpressed in breast cancer and has a strong correlation with adverse clinical outcomes [46]. mTORC2, on the other hand, phosphorylates ACLY at Ser455, enhancing its activity and promoting de novo fatty acid synthesis in breast cancer [37]. STK11/LKB1 is another pathway related to fatty acid metabolism. This tumor suppression pathway controls AMPK, which phosphorylates ACC and blocks de novo synthesis of fatty acids in breast cancer [47]. AMPK also suppresses the PI3K/Akt/mTOR signaling pathway, indirectly impacting de novo synthesis of fatty acids [48]. Additionally, c-Myc was reported to collaborate with SREBP1 to promote fatty acid de novo synthesis in various types of cancers, including breast cancer. The induction of c-Myc increases the mRNA expression of de novo fatty acid synthesis-related genes, along with the corresponding proteins, which are essential for tumorigenesis [49]. In summary, these key pathways and signaling molecules contribute to the aberrant upregulation of de novo fatty acid synthesis in breast cancer, highlighting potential therapeutic targets for intervention.

#### 3.1.2. Fatty Acid Uptake Reprogramming

Although breast cancer cells exhibit a higher propensity for de novo synthesis of fatty acids, the uptake of exogenous fatty acids is also essential for cancer cell growth and survival [50]. Breast cancer cells primarily acquire exogenous fatty acids from surrounding adipocytes. Invasive breast cancer cells significantly influence these adipocytes, leading to modifications in their phenotypes and biological characteristics, which reflects their classification as cancer-associated adipocytes (CAAs). CAAs engage in a dynamic exchange of metabolites with breast cancer cells, specifically releasing fatty acids that serve as material and energy sources for the tumor [51,52]. Therefore, elucidating the fatty acid uptake process in breast cancer may contribute to the development of novel strategies for breast cancer treatment.

Two principal pathways facilitate the uptake of fatty acids by breast cancer cells: the CD36 transport process is assisted by FABP4, as well as the endocytic uptake of LDL through LDLR (Figure 1). Gyamfi et al. found that CD36 and FABP4 expression levels are elevated in breast cancer patients [53]. Another study revealed that CD36-positive cells, termed metastasis-initiating cells (MICs), possess a unique ability to initiate metastasis. CD36 inhibition significantly reduces breast cancer metastasis [54]. LDLR overproduction is another important mechanism by which breast cancer cells obtain more essential fatty acids. Studies have indicated that elevated LDLR expression in human breast cancers correlates with reduced recurrence-free survival rates [55]. These findings underscore the importance of fatty acid uptake in the progression of breast cancer.

Two regulatory pathways involving CD36 and FABP4 have also been identified. The first pathway involves the phosphorylation of STAT3, which binds to the CD36 promoter, inducing its transcription and promoting fatty acid uptake in breast cancer cells [53]. The second regulatory mechanism involves the interaction of the nuclear receptor PPARγ with Nur77. This interaction recruits the ubiquitin ligase Trim13, targeting Nur77 for degradation, thereby suppressing the uptake of exogenous fatty acids. This is achieved by recruiting the SWI/SNF complex and HDAC1 to transcriptionally repress CD36 and FABP4 expression in breast cancer cells [56]. LDLR regulation also occurs through two major pathways. First, SREBP2 upregulates LDLR expression, enhancing LDL uptake [57]. Additionally, LXR is involved in regulating LDLR, whose activation leads to the upregulation of inducible degrader of LDLR (IDOL), an E3 ubiquitin ligase that triggers the lysosomal degradation of LDLR [58]. It is noteworthy that cholesterol, a prominent component of LDL, is also affected by these regulatory pathways. Therefore, LDLR regulation impacts both fatty acid and cholesterol levels in breast cancer.

#### 3.1.3. FAO Reprogramming

FAO is an important source of energy for cells. FAO upregulation is a common alteration detected in various types of cancer. Beyond providing energy, FAO has additional functions, including the production of NADPH, an essential reducing agent. The acetyl-CoA generated by FAO enters the TCA cycle to form citrate, which can be oxidized to α-ketoglutaric acid by isocitrate dehydrogenase, resulting in NADPH production [59]. Furthermore, FAO has been linked to epithelial–mesenchymal transition (EMT) in breast cancer. Metastatic breast cancer cells require elevated levels of FAO to manage the metabolic stress associated with metastasis [60]. Additionally, the upregulation of FAO-related genes can confer resistance to certain drugs, including tamoxifen [61].

In breast cancer, enzymes associated with FAO are often overexpressed, and inhibiting these enzymes can delay cancer growth (Figure 1). For example, the ACSL family of enzymes plays a critical role in breast cancer cells. Wang et al. reported that hepatitis B interaction protein interacts with the ACSL1 promoter and activates the transcription factor Sp1 to upregulate ACSL1 in breast cancer [62]. As another member of the ACSL family, ACSL3 has been shown to stimulate FAO and promote metastasis in TNBC, with homozygous deletion of qACSL3 being associated with poor prognosis in TNBC patients [63,64]. ACSL4 expression is associated with aggressive phenotype in breast cancer cells [65]. Additionally, several FAO-related enzymes are believed to influence the survival and proliferation of breast cancer cells. In an orthotopic patient-derived xenotransplantation TNBC model, CPT1B was shown to promote stemness and chemoresistance in breast cancer cells [66]. Other research suggested CPT1A as one of the eight growth-dependent genes in the luminal subtype of breast cancer [67]. Knockdown of ACOX2-i9, a variant of the ACOX2 enzyme found in a subgroup of human breast cancers, was shown to decrease cell viability [15]. Overall, breast cancer cells rely on the upregulation of FAO to secure sufficient energy for survival and growth [67].

The regulatory mechanism of FAO in breast cancer has attracted considerable attention (Figure 2), with PPARα identified as a major regulator. A substantial body of evidence has demonstrated that the PPARα agonist clofibrate enhances the biological activities of ACSL1 and CPT1 enzymes, thereby facilitating FAO in breast cancer cells. Conversely, clofibrate reduced ACOX1 expression in breast cancer cells, thereby exerting an opposing effect. Consequently, further investigation is required to elucidate the role of PPARα in breast cancer. Three additional regulatory pathways associated with FAO have also been identified. The first pathway is c-Myc/PGC-1β/ERRα signaling: research has shown that inhibiting FAO enzymes can block breast cancer progression driven by c-Myc [68]. The second is JAK/STAT3 signaling, which was shown to regulate CPT1 in breast cancer cells [66]. The third is the CD155/CD96/Src/STAT3/Opa1 pathway, which enhances FAO in breast cancer stem cells (BCSCs) [69]. FAO provides resources involved in mitochondrial oxidative phosphorylation, which is responsible for generating energy and reactive oxygen species (ROS) in BCSCs, processes for the maintenance of cancer stemness, and the development of chemoresistance [70,71]. By targeting these regulatory pathways, it may be possible to disrupt energy production in breast cancer cells, thereby inhibiting tumor progression.

### 3.2. Triglyceride Metabolism Reprogramming

#### 3.2.1. Triglyceride Synthesis Reprogramming

Triglycerides serve as the primary storage form of lipids, indicating that their accumulation is crucial for meeting the high energy and substrate demands of breast cancer cells [72]. Consequently, the overexpression of enzymes involved in triglyceride synthesis, e.g., GPAT2, LPIN1, and DGAT, is a common alteration in breast cancer (Figure 1). GPAT2, particularly, is significantly upregulated in breast cancer cells, which is associated with poor clinical outcomes [73,74]. The GPAT2 gene, classified as a cancer/testis gene, plays a role in the biogenesis of P-element Induced Wimpy Testis (PIWI)-interacting RNAs (piRNAs) in germline stem cells [75]. GPAT2 silencing alters the small non-coding RNA landscape, resulting in a more differentiated phenotype of breast cancer cells [76]. LPIN1, a phosphatidic acid phosphatase, is another key enzyme involved in triglyceride synthesis. He et al. found that LPIN1 is overexpressed and associated with a poor prognosis in TNBC [77]. The lipin-1 gene, which regulates LPIN1 synthesis, is targeted by tumor suppressor p53. P53 induces lipin-1 expression in response to conditions such as glucose deprivation, oxidative stress, and DNA damage [78]. Furthermore, the proto-oncogene Src mediates LPIN1 phosphorylation on tyrosine residues, which is strongly associated with malignancy and poor prognosis in breast cancer [79]. DGAT-1, a major enzyme in the synthesis of triglycerides, has been observed to be overexpressed in breast cancer cells. This contributes to the storage of lipids and the process of EMT, which in turn leads to a more aggressive breast cancer phenotype [80]. DGAT is also responsible for ferroptosis suppression by sequestering excessive PUFA, which diminishes the impact of free radicals on cancer cells [81]. Although current research has elucidated part of the regulatory mechanisms governing triglyceride and phospholipid syntheses, further investigation is needed to fully understand the significance of these processes in breast cancer.

#### 3.2.2. Triglyceride Breakdown Reprogramming

Triglyceride breakdown exhibits dual roles in breast cancer. On one hand, fatty acids produced through triglyceride breakdown promote breast cancer progression. Studies have shown that MAGL and ATGL, enzymes associated with the triglyceride breakdown process, are highly expressed in aggressive breast cancer cells, facilitating cancer migration, invasion, survival, and growth [82,83]. On the other hand, triglyceride breakdown reduces triglyceride storage. Inhibition of triglyceride degradation is believed to potentially promote breast cancer metastasis and proliferation. For instance, G0/G1 conversion gene 2 (G0S2), an ATGL inhibitor, is highly expressed in triple-negative breast cancer and associated with the invasive phenotype and poor prognosis [84]. Specifically, G0S2 induces the synthesis of integrin α5β1, which activates FAK-Src and ERK1/2 pathways, inhibits the hippo pathway, and eventually leads to cell proliferation [85]. Thus, triglyceride breakdown plays a complex role in breast cancer, and further research is required to elucidate how this process is altered in breast cancer patients.

### 3.3. Phospholipid Metabolism Reprogramming

Phospholipids are essential components of cell membranes. Their upregulation meets the increasing demand for membrane synthesis in breast cancer cells. GPLs and SPLs are two main types of phospholipids with different biological functions and distinct roles in breast cancer progression.

Li et al. demonstrated that overexpression of PI:PI3KC2α in breast cancer cells leads to increased cell migration, invasion, and metastasis. The mechanism involves the recruitment of PI3KC2α to focal adhesions, where it produces phosphatidylinositol 3,4-bisphosphate. This lipid molecule regulates rat sarcoma (R-RAS) inactivation at focal adhesions, ultimately driving metastasis in breast cancer [86]. PS is predominantly localized in the inner membrane leaflet [87]. In the tumor microenvironment, PS exposure on both tumor and immune cells has been shown to result in immune suppression, promoting tumor growth and metastasis [88]. It is notable that GPLs are predominantly unsaturated, comprising primarily PUFAs. Therefore, aberrant metabolism of GPLs is significantly associated with ferroptosis in breast cancer cells.

Cer and S1P are two key molecules in SPL metabolism. Studies reveal that enzymes involved in Cer synthesis pathways, including SPT, CerS6, and DES1, are highly expressed in breast cancer cells. These enzymes are also associated with a more aggressive and metastatic breast cancer phenotype [89,90,91]. The signaling pathway involving S1P is critical for epidermal growth factor receptor (EGFR) transactivation, which induces breast cancer migration, proliferation, and cell survival [92]. Additionally, S1P regulates CSC survival via STAT1 [93]. S1P also interacts with several intracellular targets that may play significant roles in cancer, including TNF receptor-associated factor 2 (TRAF2), HDAC, and human telomerase reverse transcriptase (hTERT) [94,95,96]. Numerous studies have reported high expression levels for SPHK1 and SP1 in breast cancer tissues, which are closely associated with poor prognosis and lower survival rates [97,98].

### 3.4. Cholesterol Metabolism Reprogramming

Recent studies have highlighted a correlation between cholesterol metabolism and breast cancer development, although the precise role of cholesterol remains uncertain. On the one hand, numerous reports have demonstrated that enzymes implicated in cholesterol synthesis exhibit elevated expression in breast cancer cells. For instance, the enzyme HMGCS1, which catalyzes the synthesis of HMG-CoA, is upregulated in both luminal and basal breast cancer subtypes and is associated with breast cancer stemness [99]. Statins that inhibit HMG-CoA reductase decrease breast cancer risk [100]. Additionally, long non-coding RNA 30 (lncRNA30) stabilizes the mRNA of SQLE, thereby enhancing cholesterol synthesis and maintaining breast cancer stem cell features, making it a promising target for breast cancer treatment [101]. The NAD(P)-dependent steroid dehydrogenase-like (NSDHL) gene, which encodes sterol dehydrogenase or decarboxylase, is also significantly overexpressed in breast cancer and correlates with poor survival outcomes, particularly in triple-negative breast cancer cases [102,103]. Conversely, multiple clinical trials have reported a negative or negligible correlation between cholesterol levels and breast cancer [104,105,106,107]. While these results may seem controversial, researchers generally agree on cholesterol’s potential impact on breast cancer incidence and outcomes. Given that cholesterol is a critical component of various cellular membranes, its rapid acquisition may be essential for cancer cells that need to synthesize new membranes.

The relationship between cholesterol and breast cancer remains complex, with several hypotheses attempting to explain it. Understanding how high cholesterol levels affect breast cancer pathogenesis is challenging due to the intricacies of cholesterol regulation. SREBP2 and LXR are two major regulators with exactly opposite functions. SREBP2 upregulates the genes responsible for cholesterol synthesis, including HMGCR and SQLE [57]. LXR-mediated induction of ABC enhances the reverse cholesterol transport from the plasma membrane to the ER, which prevents SREBP2 maturation and inhibits cholesterol synthesis in tumor cells [108]. In addition, many intermediates in the cholesterol metabolic pathway also play roles in breast cancer, further complicating the relationship [109]. For instance, farnesyl pyrophosphate, an intermediate in the mevalonate pathway, can facilitate the isoprenylation of small G proteins, which in turn promotes the proliferation and migration of cancer cells [110]. Conversely, certain factors associated with breast cancer development also affect cholesterol levels. A prime example is estrogen. Cholesterol not only affects breast cancer incidence and outcomes but also interacts with other factors, including triglyceride, estrogen, dietary components, and so on, to form a complex regulatory network [111,112].

### 3.5. The Role of Histone Modification by Acetyl-CoA in Breast Cancer

Acetyl-CoA serves as a cofactor in histone modification. A recent study demonstrated that acetyl-CoA catalyzes histone H3 lysine 27 acetylation (H3K27ac) in MICs, which is essential for EMT in breast cancer. This indicates that increasing acetyl-CoA levels in breast cancer cells promotes metastasis [113]. Numerous additional studies support this conclusion. For instance, integrin alpha-2 (ITAG2) was shown to enhance ACLY expression, leading to increased levels of acetyl-CoA and enhanced breast cancer stemness and metastasis [114]. ACC overexpression has been shown to be a significant factor in promoting de novo fatty acid synthesis and thus facilitating tumor development. However, another study revealed that inhibition of ACC results in an increase in acetyl-CoA concentration, which results in the acetylation and activation of the transcription factor Samd2, ultimately inducing EMT. This demonstrates that ACC is a multifaceted process, with both positive and negative consequences [115]. The role of acetyl-CoA in histone modification further complicates the understanding of lipid metabolism in breast cancer.

### 3.6. The Role of Lipid Metabolism in the Immune System

The role of lipid metabolism in the immune system has emerged as a significant area of research in recent years, particularly due to its critical function in the tumor microenvironment. Numerous studies have demonstrated that lipid metabolism is reprogrammed in various types of immune cells in breast cancer, including tumor-infiltrating T lymphocytes (TILs), tumor-associated macrophages, dendritic cells, and others [116]. This metabolic reprogramming also affects the expression of immune checkpoints within the tumor microenvironment, thereby affecting both the antitumor functions of the immune system and the efficacy of immune checkpoint blockade therapy.

#### 3.6.1. Tumor-Infiltrating T Lymphocytes (TIL)

TILs are a vital component of the tumor microenvironment and exhibit considerable heterogeneity regarding their biological functions. TILs can be classified into two main clusters, including CD8+ T cells, which include effector T cells, and CD4+ T cells, which encompass regulatory T cells (Tregs), helper T cells (Ths), and other cell types [117]. A substantial body of evidence indicates that lipid metabolism reprogramming varies significantly across different TIL types.

CD8+ T cells predominantly utilize glycolysis for energy production, rather than relying on FAO, which is detrimental in a hypoxic tumor microenvironment [118]. Elevated cholesterol levels can inhibit receptor signaling in CD8+ T cells and impair their proliferation and cytokine production, leading to a reduced antitumor immune response [119]. Additionally, studies have shown that SREBP activity is suppressed in CD8+ T cells in breast cancer [120], suggesting that lipid metabolism is markedly diminished in these cells.

In contrast, Tregs and Th17 cells, which collaborate to suppress immune responses in the breast cancer microenvironment, have a notable increase in lipid metabolism [121]. One study revealed that SREBP activity is stimulated in Tregs, promoting the generation of fatty acids that facilitate their proliferation and differentiation, ultimately achieving an immunosuppressive effect in breast cancer [122]. Liu et al. reported that tumor cells and Tregs induce the overexpression of phospholipase A2, which contributes to lipid metabolism dysfunction and effector T cell senescence. Inhibition of phospholipase A2 in effector T cells prevented senescence, thereby enhancing their antitumor effects and improving immunotherapy efficacy in breast cancer mouse models [123].

Th17 cells are related to the expression of CD5L, a regulator of lipid metabolism [124]. Specifically, the absence of CD5L leads to increased amounts of cholesterol and saturated fatty acids in Th17 cells, eventually suppressing immune responses [125]. Baek et al. found that 27-hydroxycholesterol (27-HC) increased the number of polymorphonuclear neutrophils and γδ-T cells at distant metastatic sites, generating a tumor microenvironment conducive to metastasis [126]. A recent study also revealed that acetate, which is metabolized into acetyl-CoA via ACSS activity, enhances the proliferation and function of T cells in breast cancer [127].

#### 3.6.2. Tumor-Associated Macrophages (TAMs)

TAMs are the most prevalent immune cells within the tumor microenvironment, and their activation status determines their role in tumor progression. M1 macrophages are activated through the classical pathway and play a crucial role in the antitumor immune response. In contrast, M2 macrophages are activated via the alternative pathway and exhibit anti-inflammatory effects, ultimately contributing to an immunosuppressive microenvironment [128,129]. Evidence suggests M2 macrophages primarily rely on FAO for their energy supply [130]. To acquire sufficient fatty acids for energy production, M2 macrophages upregulate both de novo synthesis and uptake of fatty acids, resulting in the development of an immunosuppressive phenotype within the breast cancer microenvironment [131]. Key regulatory factors in the reprogramming of lipid metabolism in TAMs include the PPAR family and the LXR receptor. PPARγ enhances the uptake of fatty acids by upregulating CD36, enabling M2 macrophages to utilize exogenous fatty acids to meet the increasing demand for FAO [132]. In contrast, LXR reduces intracellular cholesterol levels, which can disrupt the immunosuppressive effects of TAMs and inhibit breast cancer growth [133]. Additionally, fatty acids play a role in the differentiation of M1 into M2 macrophages, further stimulating breast cancer progression [134].

#### 3.6.3. Dendritic Cells (DCs)

DCs, the primary antigen-presenting cells within the breast cancer microenvironment, are essential for inducing antibody-dependent cellular cytotoxicity (ADCC), which leads to the destruction of breast cancer cells and the activation of antitumor immunity [135]. It has been demonstrated that the antigen-presenting function of DCs can be inhibited by impaired lipid metabolism [136]. Specifically, electrophilic oxidatively truncated lipids facilitate the aggregation of heat shock protein 70 on the surface of large lipid droplets, significantly reducing antigen cross-presentation in DCs [137]. Furthermore, X-box binding protein 1 is activated by accumulated lipids and their peroxidation products, disrupting dendritic cell homeostasis and thereby affecting antitumor immunity in breast cancer [138]. Lipid accumulation in DCs is achieved through the overexpression of macrophage scavenger receptor 1 (Msr1), which stimulates the internalization of fatty acids and cholesterol [139]. The potential therapeutic utility of DCs has become a primary focus of extensive research. A recent study reported that a lack of Atg5 gene expression in DCs increases CD36 expression and lipid uptake [140]. Therefore, targeting Atg5 may become a potential tool to regulate lipid metabolism and inhibit breast cancer progression.

#### 3.6.4. Natural Killer (NK) Cells 

NK cells play a pivotal role in innate immune response, inducing non-specific cytotoxicity in the breast cancer microenvironment [141]. However, tumor-infiltrating NK cells are typically repressed, resulting in impaired antitumor activity, due to an imbalance in the expression of inhibitory receptors, such as killer-cell immunoglobulin-like receptors (KIRs), and activating receptors, such as natural cytotoxicity receptors (NCRs) [142]. It was demonstrated that lipid metabolism remodeling is significantly correlated with the functionality of tumor-infiltrating NK cells. As posited by Qin and colleagues, a diet comprising a high cholesterol content may increase the total number of NK cells and exert a positive regulatory effect in mouse models of liver cancer [143]. In a mouse model of breast cancer, NK cells demonstrated a notable lipid accumulation mediated by CD36 and CD84, which results in the downregulation of perforin- and granzyme-related genes and an inhibitory effector function [144]. However, the peroxidation of lipids causes oxidative stress and disrupts the metabolic homeostasis in NK cells, which may suppress the function of NK cells. Another study revealed that the nuclear factor-erythroid factor 2-related factor 2 (Nrf2) activator RTA-408 protects NK cells from peroxidation and maintains their antitumor effects [145]. Considering both sides of lipids in NK cells, it can be postulated that the uptake of saturated FAs can provide NK cells with lipids while avoiding lipid peroxidation, which may be a potential therapeutic strategy for breast cancer.

#### 3.6.5. Myeloid-Derived Suppressor Cells (MDSCs)

MDSCs are strong immunosuppressive cells in the tumor microenvironment. Like in other immune cells, lipid metabolism reprogramming in MDSCs is also closely related to immunosuppressive function. Tumor cells can secrete a series of irritant factors to activate the STAT3 signaling pathway, which stimulates FA uptake and oxidation in MDSCs [146]. A study showed that proteins involved in fatty acid uptake, including CD36, Msr1, and FATP, are highly expressed in MDSCs [147]. Another study revealed that loss of ΔN-terminal p63 (ΔNp63) induces lipid metabolism reprogramming in MDSCs, which contributes to decreased immune suppression in ΔNp63 iKO1 TNBC models. Thus, blocking these fatty acid uptake processes may be helpful to inhibit the immunosuppressive effect of MDSCs in breast cancer.

#### 3.6.6. Immune Checkpoints in Breast Cancer Microenvironment

Immune checkpoints are a class of immunosuppressive molecules expressed on immune cells that play significant roles in shaping the tumor microenvironment. Recent research indicates that lipid metabolism is closely associated with the expression and function of immune checkpoints. A comparative study analyzed the expression levels of key immune checkpoints, including programmed cell death protein 1 (PD-1), programmed cell death protein 1 (PD-L1), and cytotoxic T-lymphocyte-associated protein 4 (CTLA-4), between groups with high fat mass index (FMI) and low FMI. The findings revealed that the high-FMI group exhibited significantly enhanced immune checkpoint expression compared to the low-FMI group [148]. Further studies have demonstrated a correlation between lipid metabolism and PD-1/PD-L1 expression in T cells. For instance, activation of the PI3K/Akt/mTOR signaling pathway has been shown to promote PD-L1 expression, thereby enhancing immune resistance in breast cancer tissue. Additionally, a study demonstrated that the FAO enhancer fenofibrate can improve the efficacy of PD-1 blockade treatment [149]. These findings suggest that upregulation of lipid metabolism may increase the expression of various immune checkpoints, potentially enhancing the effectiveness of immune checkpoint blockade therapy in breast cancer.

## 4. Biomarkers Based on Lipid Metabolism

Lipid metabolism pathways present potential biomarkers for the diagnosis, classification, metastasis, and prognosis of breast cancer. These biomarkers can be divided into two distinct categories: firstly, lipids themselves and, secondly, lipid-related enzymes.

Numerous studies have examined the use of blood lipids as biomarkers for breast cancer, partly due to the ease of measuring blood lipid levels in clinical practice [109]. Philippe et al. discovered that a composite index combining elevated monounsaturated fatty acids and low n-6/n-3 fatty acid ratios was associated with a reduced risk of breast cancer [150]. Another clinical study compared TNBC plasma to controls, finding an obvious elevation of Cer level in TNBC plasma [151]. Triglyceride and HDL level are also reported to be associated with worse overall survival (OS) and disease-free survival (DFS) in breast cancer patients [152].

Enzymes involved in lipid metabolism are also potential biomarkers. For instance, FASN has a close relationship with breast cancer metastasized to the brain, making it a potential biomarker for breast cancer brain metastasis [41]. Another study classified triple-negative breast cancer into three distinct metabolic phenotypes based on their specific metabolic profiles, among which lipid-synthesizing triple-negative breast cancer was relatively sensitive to FASN inhibitors [153]. Overall, biomarker analysis based on lipid metabolism is a hot research area with great clinical significance. However, further basic and clinical investigations into lipid metabolism are essential to identify more reliable biomarkers.

## 5. Targeted Therapy Based on Lipid Metabolism

**Figure 3:**
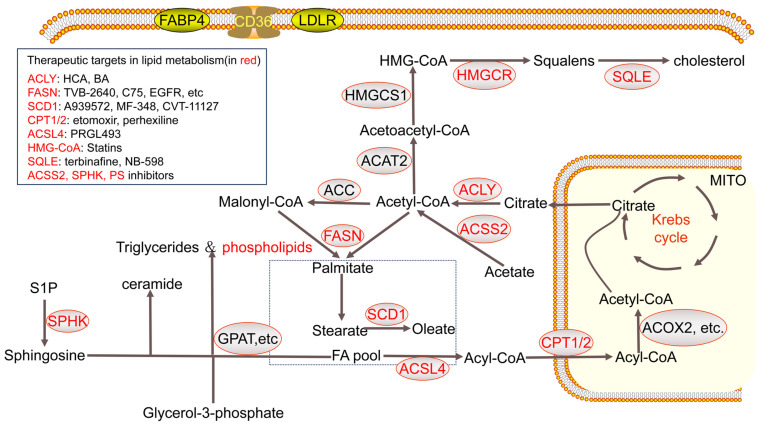
The therapeutic targets and targeted drugs in lipid metabolism. The abbreviations in this figure are shown in the ‘Abbreviations’ section.

Lipid metabolism remodeling plays a vital role in breast cancer. Therefore, targeting lipid metabolism has emerged as a potential therapeutic strategy for breast cancer patients. Several drugs that inhibit lipid metabolism enzymes have been developed for this purpose. These drugs and therapeutic targets are shown in Figure 3.

### 5.1. ACLY Inhibitors

Among the enzymes involved in lipid metabolism, ACLY has an advantage as an upstream therapeutic target, making it a preferred target for inhibiting lipid synthesis in breast cancer cells. Given the Warburg effect, cancer cells usually exhibit a greater dependency on glycolysis. ACLY becomes increasingly important as an enzyme that links glycolysis and lipid synthesis [154]. Granchi et al. summarized the application of ACLY inhibitors in anticancer strategy [155]. Among ACLY inhibitors, hydroxycitric acid (HCA) and bempedoic acid (BA) have been investigated in breast cancer cell lines. HCA has been shown to enhance the therapeutic effect of tamoxifen, reversing tamoxifen resistance [156,157]. Meanwhile, the combination of BA and CDK4/6 inhibitor palbociclib significantly reduces cell survival in breast cancer cell lines. The combined effects of palbociclib on cell proliferation and BA-associated induction of apoptosis work synergistically in breast cancer treatment [158].

### 5.2. FASN Inhibitors

FASN is a prominent target for breast cancer therapy, largely due to two distinctive characteristics: tissue distribution and enzymatic activity. Firstly, FASN demonstrates a considerably elevated level of expression in breast cancer tissue compared to normal breast tissue. Additionally, it catalyzes the terminal step of de novo fatty acid synthesis without impacting other critical components of lipid metabolism. Of all FASN inhibitors, TVB-2640 stands out as a promising candidate, having already advanced to phase II clinical trials with encouraging clinical effect and safety [159]. Other FASN inhibitors, including C75, EGFR, Fasnall, and TVB-3166, also demonstrate significant inhibitory effects on breast cancer, although they face limitations in clinical application [160,161,162,163]. Additionally, previous research focused on Chinese herbal medicines, many of which exhibit strong inhibitory effects on FASN, with some even surpassing C75 in effectiveness [164]. The increasing attention on Chinese herbal medicine in cancer treatment highlights its potential for mobilizing and regulating body functions, providing new strategies for breast cancer management.

### 5.3. SCD1 Inhibitors

SCD1 plays a crucial role in converting saturated fatty acids to monounsaturated fatty acid, which is essential for breast cancer cells to withstand lipotoxic stress [165]. Thus, SCD1 emerges as a promising target for anticancer therapy. Several SCD1 inhibitors, including A939572, MF-348, and CVT-11127, have been evaluated for treatment efficacy in breast cancer cell lines. A939572 was found to inhibit tumor cell migration induced by cancer-associated fibroblasts [166]. Another study revealed that MF-348-related inhibition of SCD1 leads to antiproliferative effects in breast cancer cells by inducing apoptosis and cell cycle arrest and preventing migration. This effect can be reversed by exogenous oleic acid, suggesting that combining SCD1 inhibitors with CD36 inhibitors may enhance tumor suppression [167]. CVT-11127 has specific functions in stimulating AMPK and reducing ACC activity, leading to enhanced suppression of de novo fatty acid synthesis and ultimately inhibiting breast cancer cell proliferation [168]. Recently, an icaritin derivative, IC2, was developed as an SCD1 inhibitor, showing potential for inducing apoptosis in breast cancer cells [43].

### 5.4. CPT1/2 Inhibitors

CPT1/2 are enzymes involved in FAO and responsible for ATP synthesis and energy supply in breast cancer cells. It was found that the CPT1 inhibitors etomoxir and perhexiline significantly reduce ATP levels and decrease cell viability in breast cancer stem cells [66]. Etomoxir has enhanced effects when used in combination with a glutaminase inhibitor, as glutamine serves as an important source for tricarboxylic acid cycle substrates, vital for energy supply in breast cancer cells. Dual inhibition of glutaminase and CPT significantly disrupts the energy supply of breast cancer cells, providing therapeutic relevance for managing drug-resistant cases [169]. Additionally, etomoxir inhibits lipid droplet formation in breast cancer cells, negatively impacting lipid accumulation and cancer cell proliferation [170].

### 5.5. Other Targeted Drugs

Drugs targeting other enzymes involved in lipid metabolism also show promising effects. For instance, ACSS2 inhibitor, as an immunoenhancing agent, enhances the effects of chemotherapies in vitro [127]. The ACSL4 inhibitor PRGL493 inhibits cell proliferation and tumor growth in a breast cancer model [171]. Statins such as HMG-CoA reductase inhibitors have been tested as cancer drugs in many studies [172]. SQLE inhibitors such as terbinafine and NB-598 lead to enhanced radiosensitivity and interrupt homologous recombination via ER stress response [173]. SPHK inhibitors synergize with doxorubicin to eradicate breast cancer CSCs and non-CSCs [93]. Phosphatidylserine-targeting antibodies obviously enhance the effects of PD-1 antibodies in anti-PD-1 immunotherapy [174].

In general, enzymes involved at various points in the lipid metabolism pathway have emerged as popular targets for breast cancer therapy. Inhibitors of these enzymes, whether used alone or in combination with other therapies, exert promising effects in breast cancer treatment. However, most of the abovementioned drugs remain at the preclinical research stage, as our understanding of lipid metabolism is still evolving. One significant challenge in the clinical application of lipid-metabolism-targeting therapies is the complexity and redundancy inherent in lipid metabolism regulation. There is considerable overlap and contradiction among the pathways involved, which may lead to compensatory mechanisms. For instance, the effects of drugs that inhibit lipogenesis can be partially mitigated by dietary lipids. Therefore, lipid-metabolism-targeting therapies need to be combined with systematic interventions to achieve better outcomes [10].

## 6. Discussion

A substantial body of research has established a correlation between lipid metabolism and breast cancer, particularly concerning several key processes, including cell proliferation, metastasis initiation, and the shaping of the immunosuppressive microenvironment. These changes ultimately contribute to the development of a more aggressive cancer phenotype and poorer clinical outcomes. However, the complexity of lipid metabolism presents significant challenges in fully elucidating its role in breast cancer. This complexity may be attributed to the following three key factors. Firstly, the multitude of lipid metabolic pathways are highly interconnected, with various processes influencing one another. Secondly, the regulation of these metabolic processes involves complex networks of enzymes, transcription factors, and signaling pathways, which can complicate the understanding of lipid metabolism in cancer biology. Thirdly, there is a dynamic exchange of intracellular and extracellular lipids, which can further influence tumor behavior and the associated microenvironment. A recent study reveals that the lipid exchange in breast cancer cells and adipocytes induces adipocyte mesenchymal transition and contributes to a tumor-friendly microenvironment [175]. Consequently, further investigation is required to provide a deeper understanding of the role of lipid metabolism in breast cancer.

Both lipids and the enzymes involved in lipid metabolism have the potential to become biomarkers for breast cancer diagnosis and prognosis. However, the lack of sufficient specificity and sensitivity limits their utility in early diagnosis and prognostic assessment. Additionally, metabolic variations among individuals and differences in lifestyle habits may lead to disparate outcomes in lipid metabolism. Notably, there is a paucity of research examining the differences in lipid metabolism across various breast cancer subtypes, making it challenging to identify biomarkers specific to these subtypes [176]. These limitations restrict the applicability of factors involved in lipid metabolism as biomarkers of breast cancer, highlighting the need for further mechanistic and clinical studies to address these issues.

Targeting lipid metabolism represents a promising strategy for the treatment of breast cancer, with several drugs currently undergoing clinical trials. However, only a limited number of these therapeutics have demonstrated notable clinical efficacy [177]. The combination of lipid-targeting therapies with dietary interventions or immunotherapy may offer a more effective means of achieving enhanced therapeutic outcomes [178]. Future studies should prioritize elucidating the specific mechanisms of lipid metabolism to facilitate the development of more effective therapeutic agents.

## 7. Conclusions

The importance of lipid metabolism remodeling in various types of malignancies, including breast cancer, has been increasingly recognized. This growing recognition, coupled with advances in research techniques, highlights its role in supporting tumor energy production, membrane biogenesis, and aggressiveness. However, the intricate mechanisms underlying the reprogramming and regulatory pathways of lipid metabolism remain to be fully elucidated. This complexity poses significant challenges to its application as a biomarker and therapeutic target. Further research is required to elucidate the intricate mechanisms of lipid metabolism for more promising clinical applications.

This review discusses the metabolism of various types of lipids, encompassing their metabolic pathways, alterations in the activities of associated metabolic enzymes, the functions of different lipid classes, and the associated regulatory proteins, genes, and pathways. The aim of this article is to summarize current findings and propose new directions for research. We hope that this review will inspire innovative research and breakthroughs in breast cancer studies. By exploring lipid-metabolism-related genes and signaling pathways, as well as their interactions with other metabolic pathways, we can identify new therapeutic targets and develop novel drugs, advancing breast cancer diagnosis and treatment into a new era.

## Abbreviations

The following abbreviations are used in this manuscript:

TNBC	Triple-negative breast cancer
LDL	Low-density lipoprotein
HDL	High-density lipoprotein
FAO	Fatty acid oxidation
ACLY	ATP citrate lyase
ACSS	Acetyl-CoA synthetase
ACC	Acetyl-CoA carboxylase
FASN	Fatty acid synthase
SCD1	Stearoyl-CoA desaturase 1
CD	Cluster of differentiation
FATP	Fatty acid transport protein
FABP	Fatty-acid-binding protein
PUFA	Polyunsaturated fatty acid
LDLR	LDL receptor
ACSL	Fatty acyl-CoA synthetase
CPT1/2	Carnitine palmitoyl transferase 1/2
ACOX	Acyl-CoA oxidase
TCA	Tricarboxylic acid
MAG	Monoacylglyceride
LPA	Lysophosphatidic acid
GPAT	Glycerol-3-phosphate O-acyltransferase
AGPAT	Acylglycerol-3-phosphate O-acyltransferase
PA	Phosphatidic acid
DAG	Diacylglyceride
DGAT	Diacylglyceride O-acyltransferase
ATGL	Adipose triglyceride lipase
HSL	Hormone-sensitive lipase
MAGL	Monoacylglyceride lipase
GPL	Glycerophospholipid
SPL	Sphingolipid
PS	Phosphatidylserine
PI	Phosphatidylinositol
PC	Phosphatidylcholine
PE	Phosphatidylethanolamine
Cer	Ceramide
SPT	L-serine:palmitoyl-CoA
CerS	Ceramide synthase
DES	Dihydroceramide desaturase
CDase	Ceramidase
SPHK	Sphingosine kinase
S1P	Sphigosine-1-phosphate
SPNS2	S1P transport spinster homolog 2
ABC	ATP-binding cassette transporters
S1PR	S1P receptor
HMG-CoA	Hydroxymethylglutaryl-CoA
HMGCS	HMG-CoA synthase
HMGCR	HMG-CoA reductase
SQLE	Squalene cyclooxygenase
LC	Liquid chromatography
MS	Mass spectromery
PI3K	Phosphoinositide 3-kinase
Akt	Protein kinase B
mTOR	Mechanistic target of rapamycin
mTORC	mTOR complex
CRTC2	CREB-regulated transcriptional factor 2
CREB	cAMP response-element-binding protein
SREBP	Sterol regulatory-element-binding protein
SCAP	SREBP cleavage-activating protein
STK11	Serine/threonine kinase 11
LKB1	Live kinase B 1
AMPK	Adenosine 5-monophosphate-activated protein kinase
c-Myc	Cellular myelocytomatosis viral oncogene
CAA	Cancer-associated adipocyte
MIC	Metastasis-initiating cell
STAT3	Signal transducer and activator of transcription 3
PPAR	Peroxisome proliferator-activated receptor
Nur77	Orphan nuclear receptor 77
SWI/SNF	Switching defective/sucrose non-fermenting complex
HDAC	Histone deacetylase
LXR	Liver X receptor
IDOL	Inducible degrader of LDLR
EMT	Epithelial mesenchymal transition
PGC	Peroxisome proliferator-activated receptor γ coactivator 1
ERR	Estrogen-related receptor
JAK	Janus kinase
BCSC	Breast cancer stem cell
ROS	Reactive oxygen species
piRNA	P-element Induced Wimpy Testis (PIWI)-interacting RNA
G0S2	G0/G1 conversion gene 2
FAK	Focal adhension kinase
ERK	Extracellular regulated protein kinase
RAS	Rat sarcoma
EGFR	Epidermal growth factor receptor
TRAF2	TNF receptor-associated factor 2
hTERT	Human telomerase reverse transcriptase
IncRNA	Long non-coding RNA
NSHDL	NAD(P)-dependent steroid dehydrogenase-like
H3K27ac	Histone H3 lysine 27 acetylation
ITAG2	Integrin alpha-2
TIL	Tumor-infiltrating lymphocyte
Treg	Regulator T cell
Th	Helper T cell
27-HC	27-Hydroxycholesterol
TAM	Tumor-associated macrophage
DC	Dendritic cell
NK	Natural killer
ADCC	Antibody-dependent cellular cytotoxicity
Msr1	Macrophage scavenger receptor 1
KIR	Killer-cell immunoglobulin-like receptor
NCR	Natural cytotoxicity receptor
Nrf2	Nuclear factor-erythroid factor 2-related factor 2
MDSC	Myeloid-derived suppressor cell
ΔNp63	ΔN-terminal p63
PD-1	Programmed cell death protein 1
PD-L1	Programmed cell death ligand 1
CTLA-4	Cytotoxic T-lymphocyte-associated protein 4
FMI	Fat mass index
HCA	Hydroxycitric acid
BA	Bempedoic acid
